# Recombinant human endostatin combined with chemotherapy for advanced squamous cell lung cancer: a meta-analysis

**DOI:** 10.1186/s12957-021-02161-1

**Published:** 2021-02-26

**Authors:** Li Feng, Zhicong Wang, Li Jing, Zhiguo Zhou, Shuai Shi, Ruoying Deng, Yibing Liu, Qingju Meng

**Affiliations:** 1 Department of Medical Oncology, Fourth Hospital of Hebei Medical University, 12 JianKang Road, Shijiazhuang, 050011 Hebei Province China; 2 Hebei Medical University, Shijiazhuang, Hebei Province China; 3 Department of Oncology, the First Hospital of Xingtai, Xingtai, Hebei Province China; 4 Department of Orthopedics, the First Hospital, 376 Shunde Road, Qiaodong District, Xingtai, 054001 Hebei Province China

**Keywords:** Recombinant human endostatin, Endostar, Endostar combined chemotherapy, Squamous cell lung cancer, Response rate, Disease control rate, Meta-analysis

## Abstract

**Background:**

This paper aims to compare the efficacy and safety of recombinant human endostatin combined with chemotherapy in patients with squamous cell lung cancer (*SqCLC*).

**Methods:**

We searched the Cochrane Library, PubMed, Embase, CNKI, Wanfang database, Metstr, VIP, and others and manually searched books and magazines until 2019 for articles about the efficacy and safety of recombinant human endostatin combined with chemotherapy in patients with *SqCLC*. A second search was conducted on the review literature. According to the criteria of the literature screen, the relevant randomized controlled trials (RCTs) and nonrandomized controlled trials (non-RCTs) of recombinant human endostatin combined with chemotherapy and chemotherapy alone in the treatment of *SqCLC* were included. After the data were extracted and analyzed, RevMan 5.3 software was used for meta-analysis for the outcome indicators. Then, heterogeneity tests and sensitivity analyses were carried out, and the publication bias of this study was tested in Stata 13.0 software. Six RCTs and eight non-RCTs were included. In total, 821 patients with *SqCLC* were included.

**Results:**

The response rate (*RR*) was 2.12 (95% CI: 1.57–2.85, *p <* 0.00001). The disease control rate (*DCR*) was 2.38 (95% CI: 1.70–3.32, *p <* 0.00001). The difference between the two groups was statistically significant. Regarding safety, the incidence rates of the adverse reactions cardiotoxicity, leukopenia, thrombocytopenia, and gastrointestinal reactions were not significantly different between the two groups (*OR* = 1.70, 95% CI: 0.79–3.68; *OR* = 0.93, 95% CI: 0.61–1.42; *OR* = 1.08, 95% CI: 0.71–1.64; *OR* = 0.86, 95% CI: 0.56–1.30, respectively).

**Conclusion:**

The combined treatment had a better therapeutic effect than chemotherapy alone. It did not increase the incidence of adverse reactions in the course of treatment.

## Background

Lung cancer is one of the leading causes of cancer-associated deaths worldwide [1]. In 2018, there were an estimated 2.1 million new lung cancer cases and 1.8 million deaths worldwide, accounting for 11.6% and 18.4% of the cancer incidence and death, respectively. It is estimated that the number of patients diagnosed with lung cancer in the world within 5 years will be 2.13 million [2]. Non-small cell lung cancer (*NSCLC*) accounts for more than 80% of lung cancers. It can be divided into adenocarcinoma, *SqCLC* and large cell cancer, and *SqCLC* accounts for approximately 1/3 of *NSCLC* [3].

Surgery is still the main treatment for early *SqCLC*. Most patients have reached the advanced stage when they are diagnosed and so have missed the best time of surgery. At present, the preferred treatment of these cases is still based on chemotherapy and radiotherapy, but the overall prognosis is poor, so the treatment of *SqCLC* is urgent. Tyrosine kinase inhibitors (TKIs), such as gefitinib, erlotinib, and icotinib, have become the first-line treatment option for patients with driver gene-positive *NSCLC*. Compared with other types of lung cancer, *SqCLC* is more closely related to smoking. Due to the low gene mutation rate, the effect of molecular targeted drug therapy is not significant at present [4].

The growth, maintenance, and metastasis of solid tumors depend on the generation of new blood vessels. Angiogenesis in tumors is a multistep and complex process that is regulated by both angiogenesis-promoting factors and angiogenesis-inhibiting factors [5]. The angiogenesis-promoting and angiogenesis-inhibiting factors in solid tumors are in a dynamic balance when the tumor is in the dormant state. Professor Folkman of Harvard Medical School proposed the strategy of blocking tumor angiogenesis, then cutting off the tumor nutrition supply to kill it in the 1970s, which pioneered a therapeutic strategy to target tumor blood vessels [6]. The emergence and application of many anti-tumor angiogenesis drugs can break that dynamic balance and break the tumor out of its dormant state to inhibit its angiogenesis. Antiangiogenic therapy has gradually become an indispensable option in current cancer treatment. For example, bevacizumab and recombinant human endostatin combined with cytotoxic drugs are commonly administered in advanced *NSCLC*. Aflibercept has been approved for the second-line treatment of advanced colorectal cancer. Multitarget kinase inhibitors such as sorafenib, sunitinib, and pazopanib are commonly administered for advanced renal cell carcinoma. Because the first-line treatment for patients with advanced *SqCLC* is still based on platinum-based doublet chemotherapy, which is associated with poor survival, rare gene mutations, and poor effects when using TKIs, the proposal of anti-tumor angiogenesis theory provides a new research direction for patients with *SqCLC*.

Recombinant human endostatin (trade name: Endostar, code: YH-16) is endostatin with an additional nine-amino-acid sequence at its N terminus. It is more stable and more potent and appeared on the market in China in 2005 [7]. It is a vascular endothelial growth factor inhibitor that can specifically act on microvascular endothelial cells, thereby inhibiting tumor angiogenesis, cutting off the nutrient supply and the metastasis channels of tumors, and eventually inducing tumoral apoptosis. Several clinical trials have suggested that Endostar combined with chemotherapy for *NSCLC*, including *SqCLC*, has brought better results than therapy with the original endostatin. Since it came to market, it has been clinically applied for many solid tumors, such as lung cancer, malignant melanoma, osteosarcoma, and nasopharyngeal carcinoma. Bevacizumab combined with cytotoxic drugs is commonly used in the treatment of *NSCLC* and colorectal cancer. However, in a phase II clinical trial to evaluate the efficacy of bevacizumab for advanced *NSCLC* patients (Study AVF0757g) [8], 6 cases of pulmonary hemorrhage occurred, of which 5 cases were in the low-dose bevacizumab group and 4 cases were life-threatening. The study showed that pulmonary hemorrhage was related to the central location of the tumor, and the clinical manifestations of squamous cell lung cancer are often of the central type. Squamous cell lung cancer was classified as the main risk factor leading to hemorrhage, so patients with *SqCLC* were excluded from the clinical trials. As a result, the efficacy and safety of anti-tumor vascular-targeted drugs, including Endostar, in the treatment of patients with *SqCLC* is still under debate. This article aims to conduct a meta-analysis of the efficacy and safety of Endostar combined with chemotherapy for advanced *SqCLC* to provide a high-quality reference for future clinical work.

## Methods

### Search strategy

The current study was conducted on the basis of the PRISMA guidelines (the Preferred Reporting Item for Systematic Reviews and Meta-Analyses) and the PRISMA extension statement on meta-analyses to obtain accurate outcomes in clinical practice. The literature retrieval was carried out online from the Cochrane Library, PubMed, Embase, CNKI, Wanfang Chinese Database, Metstr, VIP, etc., and the retrieval of books and journals were completed manually. The comprehensive literature was searched twice. The search period was from the start of each database up to 2019. The search key words for Metstr were as follows: (“Recombinant human endostatin” OR “Endostar” OR “rh-endostatin” OR “ endostatin” OR “Endu”) AND (“squamous cell lung cancer” OR “*SqCLC*” OR “lung squamous cell carcinoma” OR “squamous cell lung carcinoma”) AND “chemotherapy.” The query for PubMed was (Recombinant human endostatin[MeSH Terms]) OR (Endostar[MeSH Terms]) OR (rh-endostatin[MeSH Terms]) OR (endostatin[MeSH Terms]) OR (Endu[MeSH Terms]) AND (squamous cell lung cancer) OR (SqCLC) OR (lung squamous cell carcinoma) OR (squamous cell lung carcinoma) OR (lung cancer) AND (chemotherapy) OR (Endostar combined chemotherapy) OR (chemotherapy group alone).

### Inclusion and exclusion criteria

The following were the inclusion criteria: (a) patients: they were diagnosed with *SqCLC* or *NSCLC* (with specific *SqCLC* typing and efficacy analysis in the study) and TNM stage III or IV; (b) interventions: the patients in the control group were given chemotherapy, while the patients in the experimental group were treated with Endostar on the basis of the control treatment; and (c) outcome indicators: the total effective rate, disease control rate, and drug-related adverse reactions were extracted from the studies. Studies without a control group and studies in which patients had additional cancer, comorbidities, or organ dysfunction were excluded.

### Data extraction

Two authors independently extracted the data. Disagreements were resolved by discussion until a consensus was reached or by consulting a third author.

The content extracted from each paper included the following: (1) study information: the authors and the year of publication; (2) the number of cases in the experimental group and control group; (3) the study indicators, including PFS; (4) the baseline characteristics, including sex, age, and chemotherapy.

### Quality evaluation

The quality of the RCTs was evaluated on the modified Jadad scale [9]. This covers random sequence generation, allocation concealment, blinding, loss to follow-up, and withdrawal. The total score is seven, with four to seven indicating high-quality research and less than 4 being indicating low-quality research.

The quality scores of the non-RCTs were evaluated by the Newcastle-Ottawa Scale (*NOS*) [10].

### Statistical analysis

RevMan 5.3 statistical software was used for the meta-analysis. The chi-square test was used to test the heterogeneity of the included studies. If *p* was > 0.10 and *I*^*2*^ was not more than 50%, there was no statistical heterogeneity among the results of the studies, and a fixed-effect model (Mantel-Haenszel method) was used for analysis; otherwise, a random-effect model was used. Dichotomous variables were analyzed by estimating odds ratios (*ORs*) with 95% confidence intervals (*CIs*) for interval estimation. *p <* 0.05 was statistically significant. The publication bias of the articles was analyzed using an inverted funnel plot [11]. Begg’s and Egger’s tests were performed using Stata 13.0 software to quantify the bias. When *p* > 0.05 for Begg’s test and *p* > 0.10 for Egger’s test, no significant publication bias was suggested.

## Results

### Study inclusion and characteristics of the literature

**Fig. 1:**
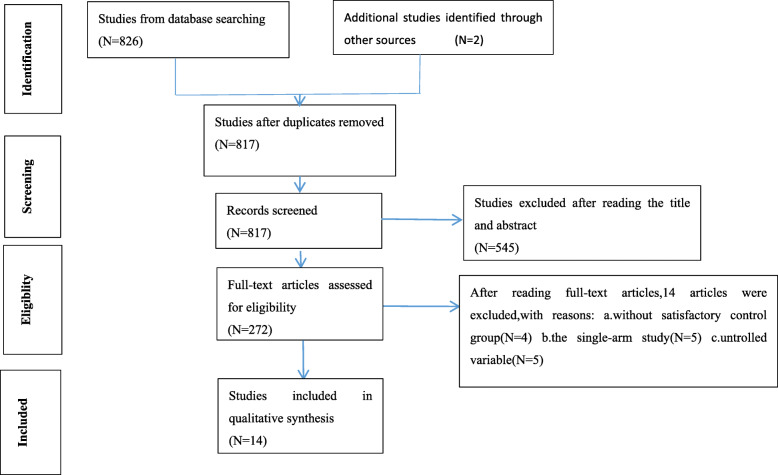
The flow diagram of the meta-analysis

From the 828 articles originally returned by the keywords, 452 articles were rejected by browsing the titles. After reading the summary of each remaining article, 272 articles remained to be read in full. Fourteen articles were included in the study after reading the full text [12–25] (Fig. 1). The characteristics of the literature are given in Table 1.

**Table 1:** Characteristics of the included studies

Studies	Number (C/E)	Age	Sex (F/M)	Regimens	PFS (C/E, months)
Wu F [12]	25/25	37–74	15/35	GNE vs GN	5.72 ± 0.89/6.90 ± 1.15
Song WC [13]	30/30	33–74	26/34	GPE vs GP	–
Wang ZF [14]	30/34	35–70	3/61	GPE vs GP	7.35 ± 0.52/13.41 ± 1.23
Chen Q [15]	19/21	65–85	–	GE vs G	3.7/4
Lv Y [16]	10/10	60–83	4/16	DPE vs DP	–
Yang L [17]	37/35	–	13/59	GPE vs GP	5.5/7.0
Zheng X [18]	40/40	60–69	29/51	GPE vs GP	6.12 ± 1.78/9.51 ± 2.15
Wang W [19]	50/50	51–56	40/60	PPE vs PP	6.3/8.4
Xun Yu [20]	14/12	46.78–65.88	–	GCE vs GC	5.1 ± 0.6/8.2 ± 1.3
Shi HL [21]	6/3	37–74	–	NPE vs NP	–
Yang L [22]	10/24	33–78	–	VPE vs VP	–
Wang QZ [23]	29/29	–	12/46	DPE vs DP	4.97/7.17
Zhang YY [24]	26/27	53–54	6/53	GPE vs GP	6.5/8.3
Hong S [25]	52/98	32–80	14/136	GPE vs GP	–

Note: The dashes represent no data;

*C* the control group, *E* the experimental group, *F* female, *M* male; *GNE* gemcitabine + nedaplatin + Endostar, *GN* gemcitabine + nedaplatin, *GPE* gemcitabine + cisplatin + Endostar, *GP* gemcitabine + cisplatin, *GE* gemcitabine + Endostar, *G* gemcitabine, *DPE* docetaxel + cisplatin + Endostar, *DP* docetaxel + cisplatin, *PPE* paclitaxel + cisplatin + Endostar, *PP* paclitaxel + cisplatin, *GCE* gemcitabine + carboplatin + Endostar, *GC* gemcitabine + carboplatin, *NPE* norvincristine + cisplatin + Endostar, *NP* norvincristine + cisplatin, *VPE* vinorelbine + cisplatin + Endostar, *VP* vinorelbine + cisplatin

### The quality assessment of studies

**Fig. 2:**
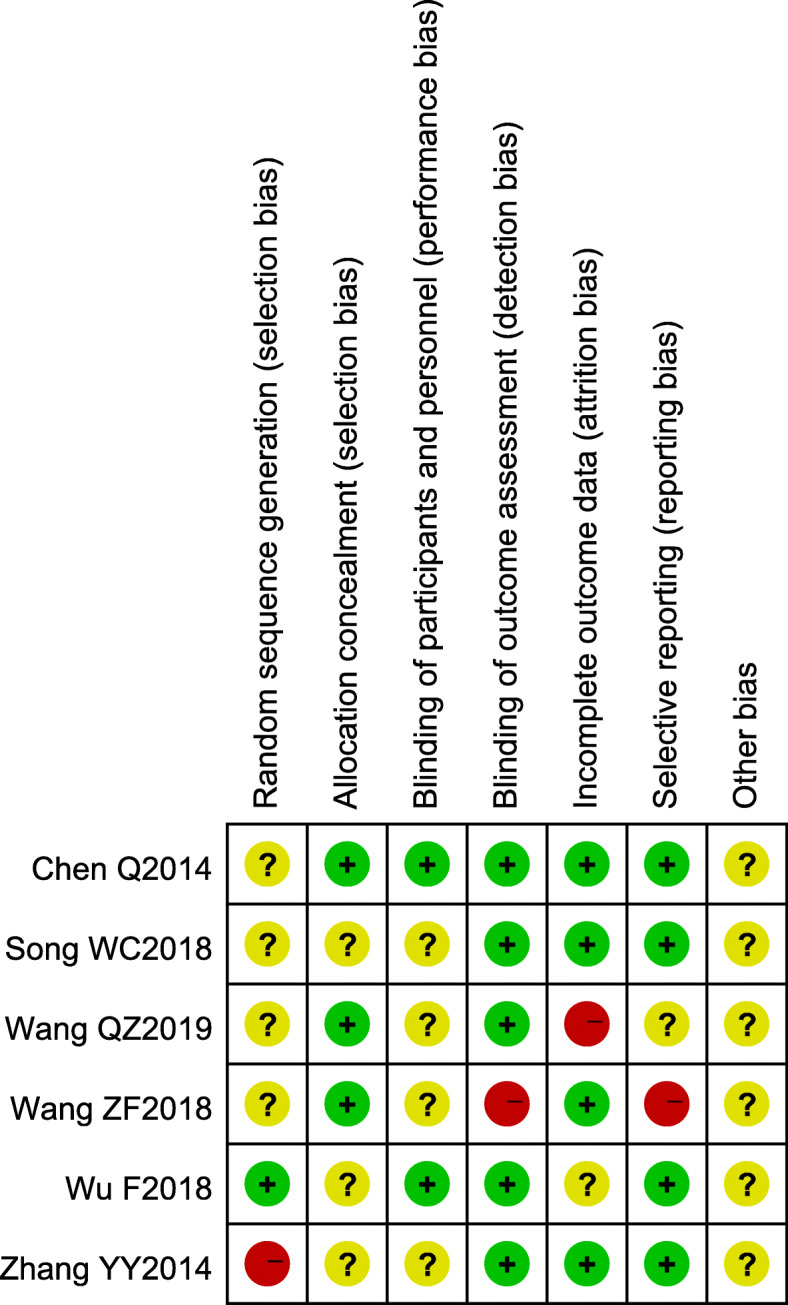
Individual risk of bias of the included RCT studies

A total of 14 articles were identified in the study, including six RCTs [12–15, 23, 24] and eight non-RCTs [16–22, 25], which were scored using corresponding quality assessment criteria. Three of six RCTs had scores lower than four points and so were evaluated as low-quality research, and the others were high-quality research. The eight non-RCTs all had scores above 5, making them high-quality studies. The quality score sheets are shown in Tables 2 and 3. The risk assessment of the RCTs was performed using the Cochrane risk assessment tool, as illustrated in Fig. 2.

**Table 2:** The quality assessment of the included RCTs

Researcher	Year	Sequence generation	Allocation concealment	Blinding	Lost and withdrawal	Jadad
Wu F	2018	Sufficient (2)	Unclear (1)	Not mentioned (1)	No (0)	4
Song WC	2018	Insufficient (0)	Insufficient (0)	Not mentioned (1)	Yes (1)	2
Wang ZF	2018	Unclear (1)	Unclear (1)	Not mentioned (1)	No (0)	3
Chen Q	2014	Unclear (1)	Unclear (1)	Not mentioned (1)	Yes (1)	4
Wang QZ	2019	Unclear (1)	Unclear (1)	Not mentioned (1)	No (0)	3
Zhang YY	2014	Unclear (1)	Unclear (1)	Not mentioned (1)	Yes (1)	4

**Table 3:** The quality assessment of non-RCTs

Researcher	Year	Population selection	Comparability between groups	Outcome data	Score
Lv Y	2015	2	2	3	7
Yang L	2018	1	2	3	6
Zheng X	2018	2	2	3	7
Wang W	2017	2	2	3	7
Xun Yu	2018	1	2	3	6
Shi HL	2004	2	2	3	7
Yang L	2005	1	2	3	6
Hong S	2016	2	2	3	7

### The clinical efficacy and safety

**Fig. 3:**
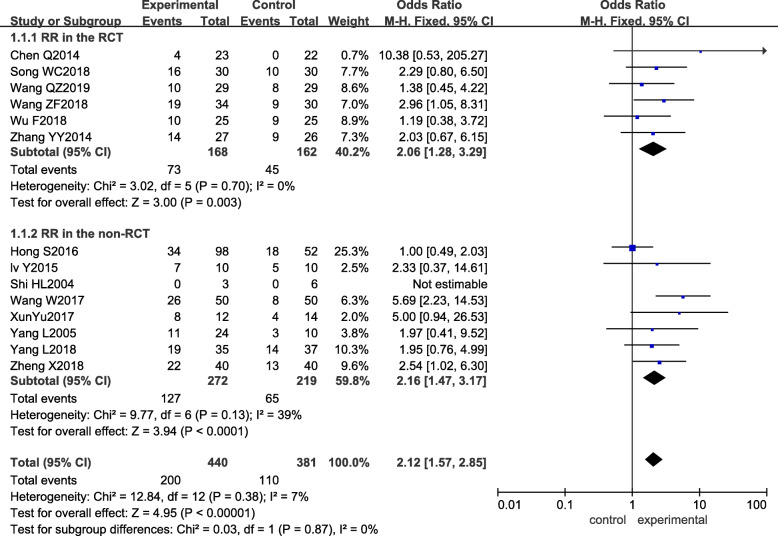
Forest plot of the experimental group vs the control group for response rate

All studies reported the response rate (*RR*). There was no significant heterogeneity between studies, so a fixed-effect model was used for meta-analysis (*p =* 0.38, *I*^*2*^ = 7%). The difference in *RR* between the experimental group and the control group was statistically significant (*OR*_mixed_ = 2.12, 95% CI: 1.57–2.85, and *p <* 0.00001), the *RR* of the experimental group being higher than that of the control group. The results of the subgroup analysis were the same as those obtained in the RCTs and non-RCTs, as shown in Fig. 3.

**Fig. 4:**
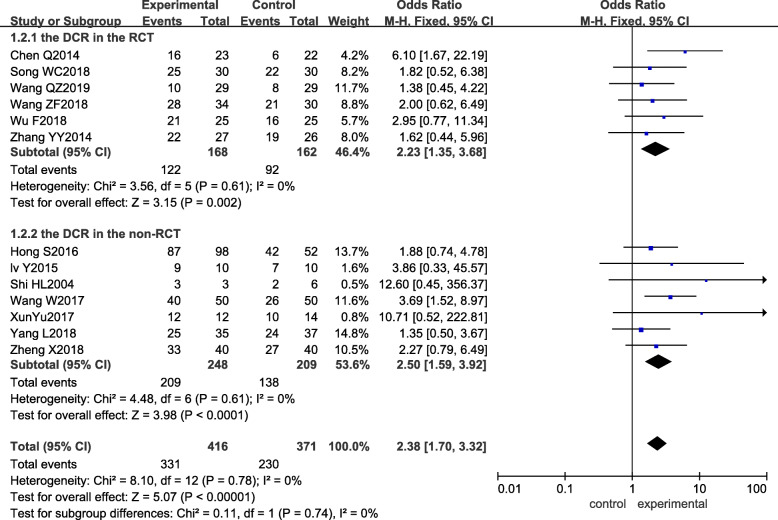
Forest plot of the experimental group vs the control group for disease control rate

Among the 14 studies included, 13 studies [12–21, 23–25] reported the disease control rate (*DCR*) as an outcome indicator. The result of the heterogeneity test showed that *I*^*2*^ was 0%, and *P* was 0.78, which indicated that there was no significant heterogeneity among the studies. Meta-analysis with the fixed-effect model revealed that *OR*_mixed_ = 2.38, 95% CI: 1.70–3.32, and *p <* 0.00001, suggesting that there was a significant difference between the two groups. The experimental group showed a better *DCR* than the control group. Subgroup analysis showed that both the RCT and non-RCT subgroups had higher *DCRs*, as shown in Fig. 4.

Adverse reactions (gastrointestinal reactions, cardiotoxicity, liver and kidney function damage, fatigue, leukopenia, thrombocytopenia, etc.) were also analyzed by meta-analysis. The fixed-effect model was used for the analysis of overall adverse event rates. The *p* values were greater than 0.05, as shown in Table 4. The incidence of adverse reactions in the two groups was almost the same, which was basically consistent with the findings of domestic and foreign studies [26, 27]. The incidence of grade III and IV adverse reactions was mentioned in four studies [12, 13, 23, 25], and the difference between the experimental group and the control group was not statistically significant (*p* > 0.05), further suggesting that Endostar is safe.

**Table 4:** The meta-analysis of adverse reactions

Adverse reaction	Number of included studies	Heterogeneity		Outcome model	OR	95% CI	*p*
		*P*	*I*^2^				
Gastrointestinal reactions	8 [12–14, 16–19, 24]	0.83	0%	Fixed-effect model	0.86	(0.56,1.30)	0.46
Cardiotoxicity	5 [12–14, 17, 24]	0.68	0%	Fixed-effect model	1.70	(0.79,3.68)	0.18
Liver and kidney function damage	4 [12, 17, 18, 24]	0.75	0%	Fixed-effect model	0.77	(0.40,1.50)	0.45
Fatigue	5 [13, 14, 17, 18, 24]	0.59	0%	Fixed-effect model	1.06	(0.60,1.87)	0.85
Leukopenia	6 [13, 14, 17–19, 24]	0.99	0%	Fixed-effect model	0.93	(0.61,1.42)	0.74
Thrombocytopenia	7 [12–14, 17–19, 24]	0.99	0%	Fixed-effect model	1.08	(0.71,1.64)	0.72

### Sensitivity analysis

**Fig. 5:**
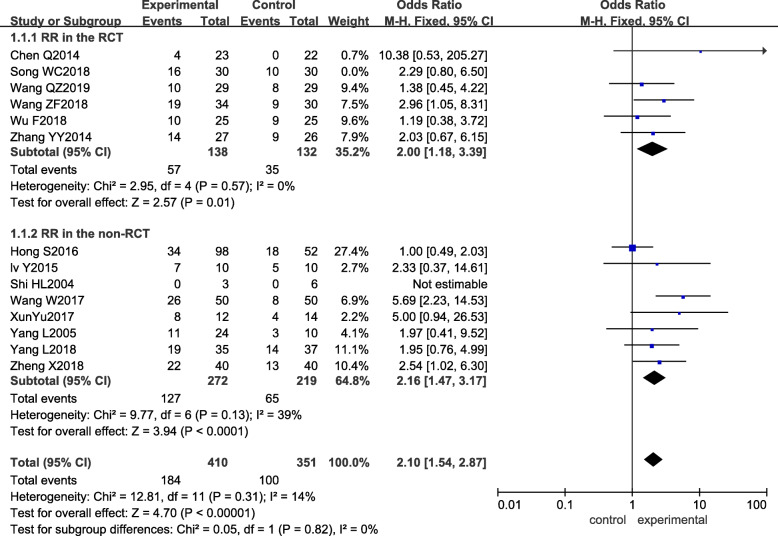
Sensitivity of the experimental group vs the control group for response rate

**Fig. 6:**
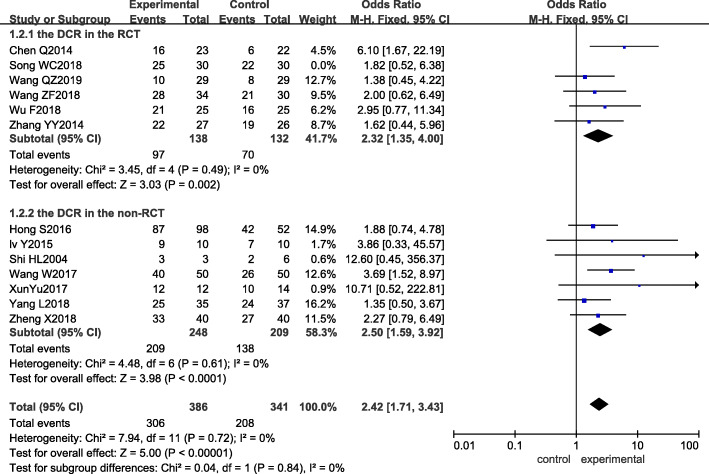
Sensitivity of the experimental group vs the control group for disease control rate

Sensitivity analysis was performed next. By removing each included study one by one and carrying out a pooled analysis, the obtained pooled effect *OR* and 95% CI still suggested that the experimental group had better *RR* and *DCR*, and the heterogeneity after individual study removal was similar to before, as seen in Tables 5 and 6 in detail. According to the results of the quality evaluation of the included studies, the *Q* value and *I*^*2*^ of the response rate and disease control rate after removal of Song WC [13], which had lower scores, were *p =* 0.31, *I*^*2*^ = 14%, and *p =* 0.72, *I*^*2*^=0%, respectively, showing no significant change in heterogeneity, meaning the results of this study were robust, as shown in Figs. 5 and 6.

**Table 5:** Sensitivity analysis of response rate

Removed study	*p*	*I*^2^ (%)	Odds ratio (95% CI)
–	0.38	7	2.12 (1.57, 2.85)
Chen Q [15]	0.39	6	2.06 (1.53, 2.78)
Song WC [13]	0.31	14	2.10 (1.54, 2.87)
Wang ZF [14]	0.34	11	2.06 (1.51, 2.80)
Wu F [12]	0.38	7	2.21 (1.62, 3.01)
Wang QZ [23]	0.34	10	2.19 (1.61, 2.98)
Zhang YY [24]	0.30	14	2.13 (1.56, 2.89)
Lv Y [16]	0.30	14	2.11 (1.56, 2.86)
Shi HL [21]	0.38	7	2.12 (1.57, 2.85)
Wang W [19]	0.72	0	1.88 (1.37, 2.58)
Xun Yu [20]	0.38	6	2.06 (1.52, 2.79)
Yang L [22]	0.30	14	2.12 (1.57, 2.88)
Yang L [17]	0.31	14	2.14 (1.56, 2.92)
Zheng X [18]	0.32	13	2.07 (1.51, 2.84)
Hong S [25]	0.73	0	2.50 (1.80, 3.47)

**Table 6:** Sensitivity analysis of disease control rate

Removed study	*p*	*I*^2^ (%)	Odds ratio (95% CI)
–	0.78	0	2.38 (1.70, 3.32)
Chen Q [15]	0.89	0	2.21 (1.56, 3.13)
Song WC [13]	0.72	0	2.42 (1.71, 3.43)
Wang ZF [14]	0.71	0	2.41 (1.70, 3.42)
Wu F [12]	0.72	0	2.34 (1.66, 3.31)
Wang QZ [23]	0.78	0	2.51 (1.76, 3.56)
Zhang YY [24]	0.73	0	2.44 (1.73, 3.45)
Lv Y [16]	0.72	0	2.35 (1.68, 3.30)
Shi HL [21]	0.79	0	2.32 (1.66, 3.25)
Wang W [19]	0.81	0	2.20 (1.53, 3.16)
Xun Yu [20]	0.79	0	2.30 (1.64, 3.23)
Yang L [17]	0.81	0	2.55 (1.79, 3.65)
Zheng X [18]	0.70	0	2.83 (1.84, 4.35)
Hong S [25]	0.72	0	2.39 (1.68, 3.40)

### Publication bias

**Fig. 7:**
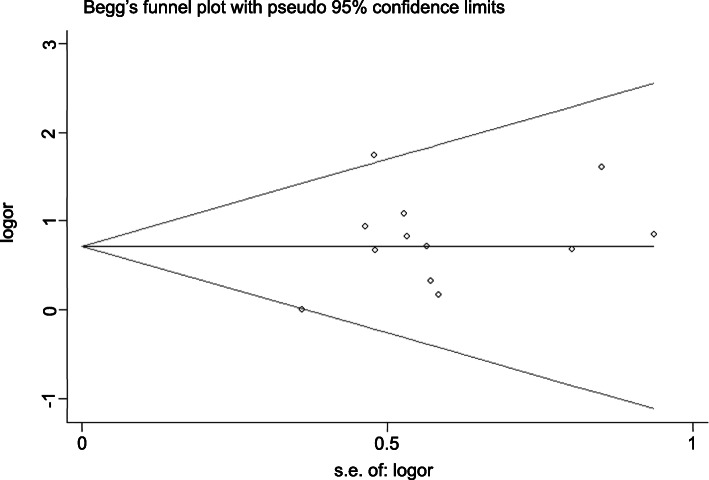
Begg’s funnel plot of the experimental group vs the control group for response rate

**Fig. 8:**
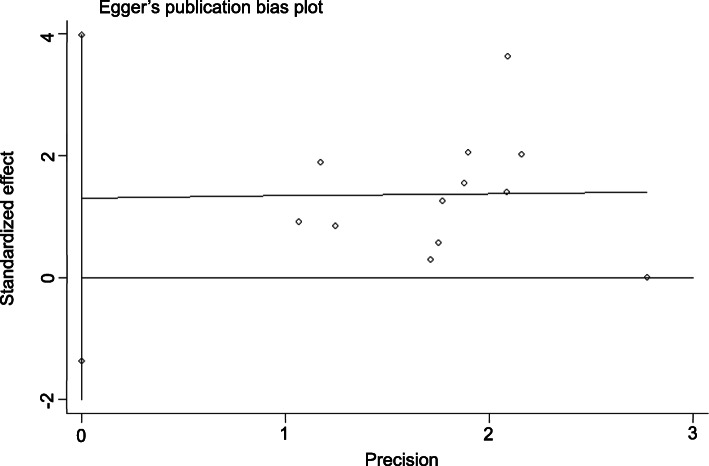
Egger’s publication bias plot of the experimental group vs the control group for response rate

**Fig. 9:**
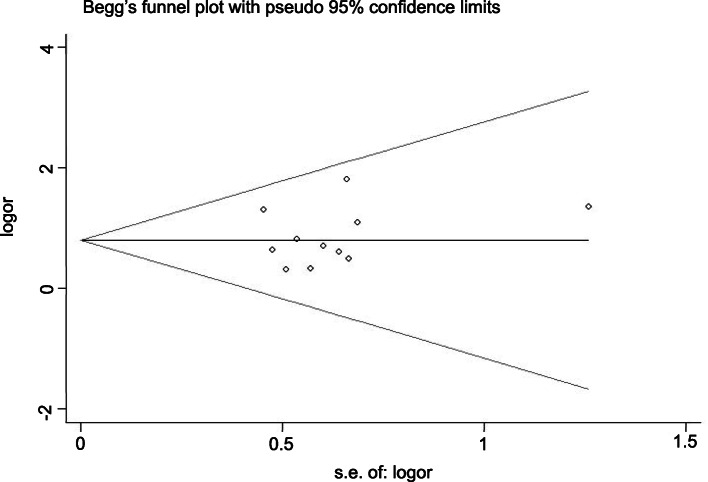
Begg’s funnel plot of the experimental group vs the control group for disease control rate

**Fig. 10:**
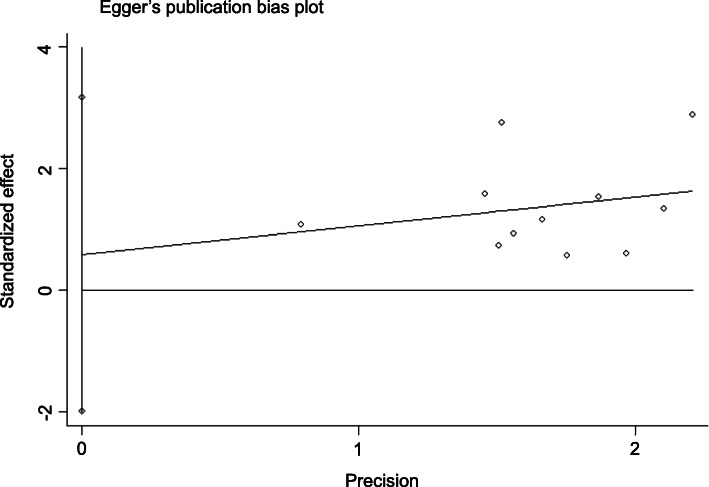
Egger’s publication bias plot of the experimental group vs the control group for disease control rate

Publication bias analysis was performed using Stata 13.0 software. Begg’s test showed *p =* 0.945 > 0.05, and Egger’s test showed *p =* 0.302 > 0.10, suggesting that there was no significant publication bias in the analysis of *RR*. The *DCR* had *p =* 0.436 > 0.05 by Begg’s test and *p =* 0.615 > 0.10 by Egger’s test, suggesting that there was no significant bias in the analysis of *DCR*. We can conclude that the publication bias of this paper is small and that the included studies are comprehensive (Figs. 7, 8, 9, 10).

## Discussion

Endostar is a new biological drug independently researched and developed in China and is the first anti-tumor vascular-targeted endothelial inhibitor in the world. It was approved by the State Food and Drug Administration (SFDA) for marketing in 2005 and approved for combination with vinorelbine and cisplatin for *NSCLC* in 2016. Studies have shown that Endostar can specifically inhibit the migration of endothelial cells and induce the apoptosis of endothelial cells, thereby targeting the inhibition of angiogenesis [7]. In China, many scholars have carried out meta-analyses on the efficacy of Endostar combined with chemotherapy for *NSCLC*. However, the biological behaviors of *SqCLC* and adenocarcinoma are completely different. There have been few studies on the meta-analysis of Endostar combined with chemotherapy in squamous cell lung cancer at home or abroad, so we carried out a meta-analysis to estimate its outcomes. In terms of efficacy, the difference between the experimental group and the control group was statistically significant (*OR*_mixed_=2.12, 95% CI: 1.57–2.85, and *p <* 0.00001), the *RR* of the experimental group being higher than that of the control group. With regard to *DCR*, the outcome was *OR*_mixed_ = 2.38, 95% CI: 1.70–3.32, and *p <* 0.00001. The results were not significantly heterogeneous. According to sensitivity analysis, the results of this study are stable, and the publication bias is small. The included studies were comprehensive, so this meta-analysis provides a strong evidence-based foundation for the treatment of patients with advanced *SqCLC* in clinical practice. Common adverse reactions, consisting of myelosuppression, fatigue, cardiotoxicity, gastrointestinal reactions, and so forth, were mentioned, but there was no statistically significant difference between the experimental group and the control group. Five studies mentioned the condition of cardiotoxicity. A total of 302 patients were included in the meta-analysis of this condition under a fixed-effects model, and the OR was 1.70 (95% CI: 0.79–3.68, *p =* 0.18) (Table 4). Although the difference was not significant, it still suggested that patients with Endostar are more susceptible to cardiotoxicity. The clinical features showed increased heart rate, palpitations, changes in the T wave and ST-T segment of the electrocardiogram, etc. Therefore, more attention should be paid to the use of ECG monitoring and echocardiograms and closely monitoring patients’ heart function. Other, rare adverse reactions, such as peripheral neurotoxicity, hemorrhage, and diarrhea, are not mentioned in these studies but should not be neglected by clinicians.

Our research has some limitations. Randomized and nonrandomized controlled trials were included, and most RCT studies did not report how they created the random sequences and implemented blinding. Confusion bias inevitably arose. Endostar was administered intravenously in the two trials [15, 2 0], different from infusion pump administration in other trials. Therefore, the mode of administration, the sequence of administration, and the age and sex of patients could all have affected the result, but there was no corresponding data analysis. More factors affecting the efficacy also need to be studied. Miao M [28] suggested that the therapeutic effect, effective rate, and disease control rate after intravenous pump infusion of Endostar were all better than those after intravenous drip infusion (*p <* 0.05). Bone marrow suppression and cardiotoxicity in the pump infusion group were less than those in the intravenous drip group. In addition, the degree was alleviated. Therefore, intravenous pumping may be a better option for delivering Endostar clinically. Only two studies included in this paper were in English because Endostar has not come to any markets outside of China. This avoided the influence of gene polymorphism to some extent. Because of the limited number of studies included and the missing trials with negative results, a publication bias could be in play. Endostar, as a novel choice, still has many issues to be solved. More research with larger sample sizes and higher quality is needed to confirm that Endostar with chemotherapy can bring better efficacy and safety.

## Conclusion

The results showed that Endostar combined with chemotherapy had better efficacy than chemotherapy alone. The adverse reactions were not significantly different between the two groups.

## Data Availability

The datasets used and/or analyzed during the current study are available from the corresponding author on reasonable request.
